# LuQi Formula relieves ventricular remodeling through improvement of HIF-1α-mediated intestinal barrier integrity

**DOI:** 10.1186/s13020-023-00803-y

**Published:** 2023-07-28

**Authors:** Jirong Yan, Zhichao Xi, Jiaying Guo, Lin Xu, Xueyang Sun, Wanjing Sha, Milin Liu, Shenyu Zhao, Enrui Dai, Yu Xu, Hongxi Xu, Huiyan Qu

**Affiliations:** 1 Institute of Cardiovascular Disease of Integrated Traditional Chinese and Western Medicine, Shuguang Hospital affiliated to Shanghai University of Traditional Chinese Medicine, No. 528, Zhangheng Road, Shanghai, 201203 China; 2 School of Pharmacy, Shanghai University of Traditional Chinese Medicine, No. 1200, Cailun Road, Shanghai, 201203 China; 3 Engineering Research Center of Shanghai Colleges for TCM New Drug Discovery, Shanghai, 201203 China

**Keywords:** Ventricular remodeling, Intestinal barrier integrity, LuQi Formula, Tight junction proteins, Hypoxia inducible factor-1 alpha

## Abstract

**Background:**

Ventricular remodeling is the adaptive process in which the heart undergoes changes due to stress, leading to heart failure (HF). The progressive decline in cardiac function is considered to contribute to intestinal barrier impairment. LuQi Formula (LQF) is a traditional Chinese medicine preparation widely used in the treatment of ventricular remodeling and HF. However, the role of LQF in the impairment of intestinal barrier function induced by ventricular remodeling remains unclear.

**Materials and methods:**

Ventricular remodeling was induced in rats by permanently ligating the left anterior descending branch coronary artery, and cardiac function indexes were assessed using echocardiography. Heart and colon tissue morphology were observed by hematoxylin–eosin, Masson’s trichrome and Alcian Blue Periodic acid Schiff staining. Myocardial cell apoptosis was detected using TUNEL and immunohistochemistry. Circulatory levels of brain natriuretic peptide (BNP), intestinal permeability markers endotoxin, D-lactate and zonulin, as well as inflammatory cytokines tumor necrosis factor alpha and interleukin-1 beta were measured by Enzyme-linked immunosorbent assay. Expression levels of tight junction (TJ) proteins and hypoxia-inducible factor-1 alpha (HIF-1α) in colon tissue were detected by immunofluorescence, immunohistochemistry and western blotting. Cardiac function indexes and intestinal permeability markers of patients with HF were analyzed before and after 2–4 months of LQF treatment.

**Results:**

LQF protected cardiac function and alleviated myocardial fibrosis and apoptosis in rats with ventricular remodeling. LQF protected the intestinal barrier integrity in ventricular remodeling rats, including maintaining colonic tissue morphology, preserving the number of goblet cells and normal expression of TJ proteins. Furthermore, LQF upregulated the expression of HIF-1α protein in colon tissue. Intervention with a HIF-1α inhibitor weakened the protective effect of LQF on intestinal barrier integrity. Moreover, a reduction of HIF-1α aggravated ventricular remodeling, which could be alleviated by LQF. Correspondingly, the circulating levels of intestinal permeability markers and BNP in HF patients were significantly decreased, and cardiac function markedly improved following LQF treatment.

**Conclusions:**

We demonstrated that LQF effectively protected cardiac function by preserving intestinal barrier integrity caused by ventricular remodeling, at least partially through upregulating HIF-1α expression.

**Supplementary Information:**

The online version contains supplementary material available at 10.1186/s13020-023-00803-y.

## Background

Acute myocardial infarction (AMI) is a common ischemic myocardial injury. Pathological changes induced by AMI include myocardial cell apoptosis or necrosis, myocardial fibrosis, and impaired ventricle systolic and/or diastolic function, which contribute to the progressive pathological stage of ventricular remodeling [1]. As cardiac function deteriorates, the heart progresses into a decompensated phase, leading to heart failure (HF) with increasing rehospitalization and mortality rates over the past decade [2, 3]. HF is the terminal stage of numerous cardiovascular diseases, characterized by decreased cardiac output and disrupted systemic circulation, which results in inadequate perfusion of end organs, including intestine. Consequently, this impairment leads to compromised intestinal barrier integrity and dysbiosis in gut microbiota [4, 5]. Related studies have shown that the tight junction (TJ) proteins including zonula occludens-1 (ZO-1) and Claudin-1, are involved in the constitution of the intestinal barrier [6]. In HF rats, these proteins were significantly downregulated in the colon tissue, resulting in increased intestinal permeability and the leakage of bacterial metabolites into circulation, such as endotoxin (LPS) and D-lactate, which further contributed to systemic inflammation [7, 8]. Clinical evidence has confirmed that in patients with AMI, the enrichment of LPS and D-lactate in the blood is associated with chronic inflammation in the body [9, 10], which could further exacerbate myocardial damage [11]. Thus, a regulatory loop exists between cardiac dysfunction and intestinal barrier damage. Preclinical studies have also demonstrated the beneficial effects of preserving intestinal barrier integrity on cardiac function. For example, microbiota transplantation has been shown to restore damaged intestinal structure and suppress aged-related atrial fibrillation in rats [12]. Additionally, treatment with short chain fatty acids, known as beneficial gut metabolites, has been found to improve intestinal barrier function and reduce atherosclerosis in the ApoE^ − / −^ mice [13]. In light of these findings, maintaining the integrity of the intestinal barrier represents a novel therapeutic strategy for alleviating cardiac dysfunction resulting from ventricular remodeling [14].

LuQi Formula (LQF), a traditional Chinese medicine formula (Chinese national patent number ZL2014102933164) consisting of *Cervi Cornu, Astragali Radix, Cinnamomi Cortex, Codonopsis Radix, Carthami Flos and Descurainiae Semen Lepidii Semen*, has been widely used for treating HF clinically. LQF can significantly improve the cardiac function of HF patients, as indicated by decreased markers of cardiac dysfunction such as brain natriuretic peptide (BNP), left ventricular ejection fraction (LVEF) and left ventricular fractional shortening (LVFS) [15–17]. The cardioprotective mechanism of LQF has also been investigated in our previous preclinical study. LQF could regulate the Nod-like receptor family pyrin domain containing 3 (NLRP3) inflammasome to relieve cardiac remodeling induced by myocardial infarction in mice [18]. We previously found that LQF alleviated the intestinal injury in HF rats and inhibited the increase of pro-inflammatory factors and toxic gut metabolites in circulation, such as LPS and trimethylamine N-oxide (TMAO) [19], suggesting that LQF may act on the ‘heart-gut axis’ during HF.

Hypoxia-inducible factor-1 alpha (HIF-1α) rapidly responds to protect the physiological structure and function of the intestinal barrier when blood flow and oxygen supply are reduced [20, 21]. It has been reported that under hypoxic conditions, HIF-1α maintains the normal expression of TJ proteins and prevents the loss of goblet cells, which are responsible for secreting glycoproteins and mucins to form the intestinal mucus layer [21–23]. Deletion of HIF-1α significantly exacerbates alcohol-induced barrier dysfunction and downregulation of TJ proteins [21], while increased levels of HIF-1α in gut tissue significantly upregulate TJ proteins and enhance epithelial integrity [24]. The mechanism by which HIF-1α regulates TJ proteins is proposed to be mediated by the butyrate content in the gut [25]. Additionally, HIF-1α induces the production of intestinal trefoil factor (ITF) during hypoxia, which plays a vital role in maintaining intestinal mucosal integrity [26, 27]. Nonetheless, whether HIF-1α can attenuate HF-induced gut barrier dysfunction has not yet been reported. In the present study, we demonstrated that LQF attenuated intestinal barrier injury induced by ventricular remodeling through upregulating HIF-1α in colon tissue. Moreover, LQF further relieves ventricular remodeling caused by HIF-1α reduction. Our findings partially uncovered the mechanism of LQF in improving cardiac function during HF, and provide scientific evidences supporting LQF’s clinical application.

## Methods

### Chemicals and reagents

LQF granules containing *Cervi Cornu, Astragali Radix, Cinnamomi Cortex, Codonopsis Radix, Carthami Flos and Descurainiae Semen Lepidii Semen*, were provided by Shanghai Yangzijiang Pharmaceutical Co., Ltd, and dissolved with hot water for patients’ administration. LQF dry powder for animal experiment (1 g powder equal to 3.4 g crude drug) was dissolved in Milli-Q (RHB2S25, IKA), and stored at 4 ℃, which produced by Shanghai Chinese Traditional Pharmaceutical Technology Co., Ltd. The HPLC-Q-TOF/MS analysis data of chemical composition identification in LQF dry powder are shown in Additional file 1: Fig. S1. For LQF dry powder Benazepril hydrochloride (BNZ) (H20044840, Sinopharm) was purchased from Shanghai Xinya Drug Co., Ltd, dissolved in Milli-Q, and stored at 4 °C. 2-Methoxyestradiol (2ME) was obtained from Shanghai Yuanye Bio-Technology Co., Ltd, and was dissolved according to previously described methods [28] and stored at − 20 °C. RIPA lysis buffer (P0013C) and protease inhibitor cocktail (P1005) were obtained from Beyotime Biotechnology, China.

### Animal experiments

Male Wistar rats (160–180 g body weight) were purchased from the Laboratory Animal Center of Shanghai University of Traditional Chinese Medicine and kept under controlled temperature (20 ± 5 °C) and humidity (55 ± 15%) with a 12 h light/ 12 h dark cycle. After 1 week of adaptive rearing, ventricular remodeling was induced by permanently ligating the left anterior descending branch (LAD) coronary artery of the heart [29]. The sham group underwent the same surgical procedures without ligation. All animal experiments were conducted in compliance with relevant laboratory animal welfare and ethical norms, and the studies were approved by the ethics committee (NO. PZSHUTCM220307004).

### Echocardiography

Echocardiography was performed on rats under rapid anesthesia with 3% isoflurane and maintained anesthesia with 1.5% isoflurane to assess their cardiac function using a Vevo 2100 Imaging system (Visual Sonics Inc., Canada) equipped with a small animal ultrasound probe. The longitudinal section of the long axis of the heart was probed by placing the ultrasound probe between the 3rd and 4th intercostal space at the left sternal border. Then the key indicators of M-mode echocardiography of the mid-ventricle were recorded, including LVEF and LVFS.

### Enzyme-linked immunosorbent assay (ELISA)

The abdominal aortic blood of rats was collected, and after standing at 4 °C for 2 h, the supernatant was obtained by centrifugation. BNP (YB-BNP-Ra), LPS (YB-LPS-Ra), D-lactate (YB-DLA-Ra) and zonulin (YB-ZONULIN-Ra) in plasma, tumor necrosis factor alpha (TNF-α) (YB-TNF α-Ra) and interleukin-1 beta (IL-1β) (YB-IL 1β-Ra) in serum were detected by ELISA kits purchased from Shanghai Yu Bo Biotech Co., Ltd. Standards and samples were added to enzyme-coated plates. After being washed, the plates were incubated with avidins conjugated with horseradish peroxidase (HRP) and the substrate 3,3ʹ,5,5ʹ tetramethylbenzidine for 30 min and 15 min, respectively. The reaction was terminated by adding sulfuric acid solution, and spectrophotometric determination was performed at a wavelength of 450 nm.

### Histological staining and immunohistochemistry

The heart and colon tissues of rats were harvested immediately after euthanasia on ice and rinsed with phosphate buffer at 4 °C. The tissues were fixed in 4% paraformaldehyde (G1101, Wuhan Saiville Biotechnology Co., Ltd) for 24 h and then processed for dehydration, paraffin embedding, and sectioning to a thickness of 5 μm. The samples were stained with hematoxylin–eosin (H&E) (BH0001), Masson’s trichrome (BH0002), Alcian Blue Periodic acid Schiff (AB-PAS) (B0031), cleaved-Caspase 3 (341034, Share-Bio) and HIF-1α (GTX127309, GeneTex). Subsequently, the sections were incubated in HRP-labeled secondary antibodies (ab205718/ab5719, Abcam) of the corresponding species to the primary antibody for 1 h in the dark. The sections were then incubated in a DAB chromogenic kit (K3468, DAKO) and the nuclei were counterstained with hematoxylin. All the dyes were obtained from Wuhan Boerfu Biotechnology Co., Ltd. Finally, the sections were mounted with neutral gum, and then random fields were selected and viewed in each section for histology analysis (BX53, OLYMPUS).

### Immunofluorescence

Colon tissues of rats were isolated on ice, rinsed at 4 °C phosphate buffer, and fixed in 4% paraformaldehyde for over 24 h. After dehydration and paraffin embedding, antigen retrieval and serum blocking were performed on sections. Then, the sections were incubated overnight with Claudin-1 (sc-166338), Occludin (sc-133256) and ZO-1 (sc-33725) antibodies purchased from Santa Cruz, USA. The sections were then incubated with CY-3-labeled sheep anti-mouse antibody (F2761, Thermo Fisher Scientific, USA). The nuclei were counterstained with DAPI (C0065, Beijing Solarbio Science & Technology Co., Ltd). All sections were mounted with neutral gum and observed under an inverted fluorescence microscope (IX83, OLYMPUS). Image J software was used to semi-quantify three randomly selected regions in each slice.

### TdT-mediated dUTP nick-end labeling (TUNEL)

Heart tissues of rats were paraffin-embedded, sliced into 5 μm sections, and deparaffinized. The sections were treated with an autofluorescence quencher and 20 μg/mL of proteinase K to make the sample permeable, and washed with PBS for 5 min. The sections were incubated with TUNEL dye (C1086, Beyotime Biotechnology, China) at 37 °C for 1 h. After washing three times with PBS, the nuclei were counterstained with DAPI for 10 min, and the sections were immediately covered with autofluorescence quenching sealant. All sections were scanned using CaseView 2.0 (Pannorama 250/MIDI, 3DHISTECH, Budapest, Hungary).

### Western blotting

The frozen cell or colonic tissue was homogenized and lysed, then tissue proteins were extracted and quantified. Gel electrophoresis and antibody incubation were subsequently performed according to our earlier protocol [30]. In this study, antibodies were used to identify HIF-1α, Occludin, Claudin-1, and β-actin (A19062, ABclonal, China). The protein bands were detected using a chemiluminescence system (Beijing Sage Creation Science Co., Ltd.), and quantitatively analyzed using Image J software.

### Participants in clinical study

The study was designed according to the CONSORT 2017 STATEMENT, and approved by the Hospital Medical Ethics Committee (NO. 2020-920-129-01), adhering to the principles of the Declaration of Helsinki. The study population consisted of male and female patients (outpatients and inpatients) admitted to the Department of Cardiology of Shuguang Hospital affiliated with the Shanghai University of Traditional Chinese Medicine. All patients have signed informed consent. Inclusion criteria for the study were as follows: (1) age over 18 years; (2) diagnosis of cardiac insufficiency and New York Heart Association Functional Classification (NYHA) grade II-IV as the primary research object; and (3) voluntary participation with informed consent. Exclusion criteria were: (1) multisystem infection, tumors, connective tissue or autoimmune diseases, recent surgery or trauma; (2) moderate to severe dementia or mental illness that would hinder follow-up investigations; (3) use of antibiotics, probiotics, or hormone replacement therapy within 6 months before enrollment; and (4) participation in another study at the same time. During the treatment period, the participants received LQF granules (equivalent to 107 g of raw drug) three times a day, and no dietary restrictions were imposed during the experiment.

### Outcome measures and efficacy assessments

Blood samples were collected from the patients before and after taking LQF. Baseline blood routine tests and biochemical assays using an automated hematology analyzer (AU5800, Beckman Coulter, USA). Upon admission, BNP levels were measured using an automated immunoassay analyzer (Access2, BECKMAN COULTER). The ELISA kits were used to measure the levels of LPS (EC64405, Xiamen Bioendo Technology Co., Ltd.), D-lactate (YB-DLA-Hu, Shanghai Yu Bo Biotech Co., Ltd.) and zonulin (YB-ZONULIN-Hu, Shanghai Yu Bo Biotech Co., Ltd.) before and after the LQF intervention. Cardiac function indicators, such as LVEF, LVFS, LVSV, LVEDV and LVESV were measured before and after LQF administration using the EPIQ 7 ultrasound system for radiology (PHILIPS).

### Statistical analysis

The data were analyzed and plotted using GraphPad Prism 6 software. Paired-sample t-tests were used for two-group comparisons for the data followed a normal distribution and had aligned variances, while one-way ANOVA is used for multi-group ANOVA for three or more groups. For the data that did not follow a normal distribution and had uneven variances, logarithm transformation was performed and the normality and variance homogeneity were retested. If the test is passed, the above statistical method is used. And all data are expressed using mean ± standard deviation. Values of *p < 0.05, **p < 0.01 and ***p < 0.001 indicated statistically significant.

## Results

### *LQF relieves ventricular remodeling *in vivo

**Fig. 1:**
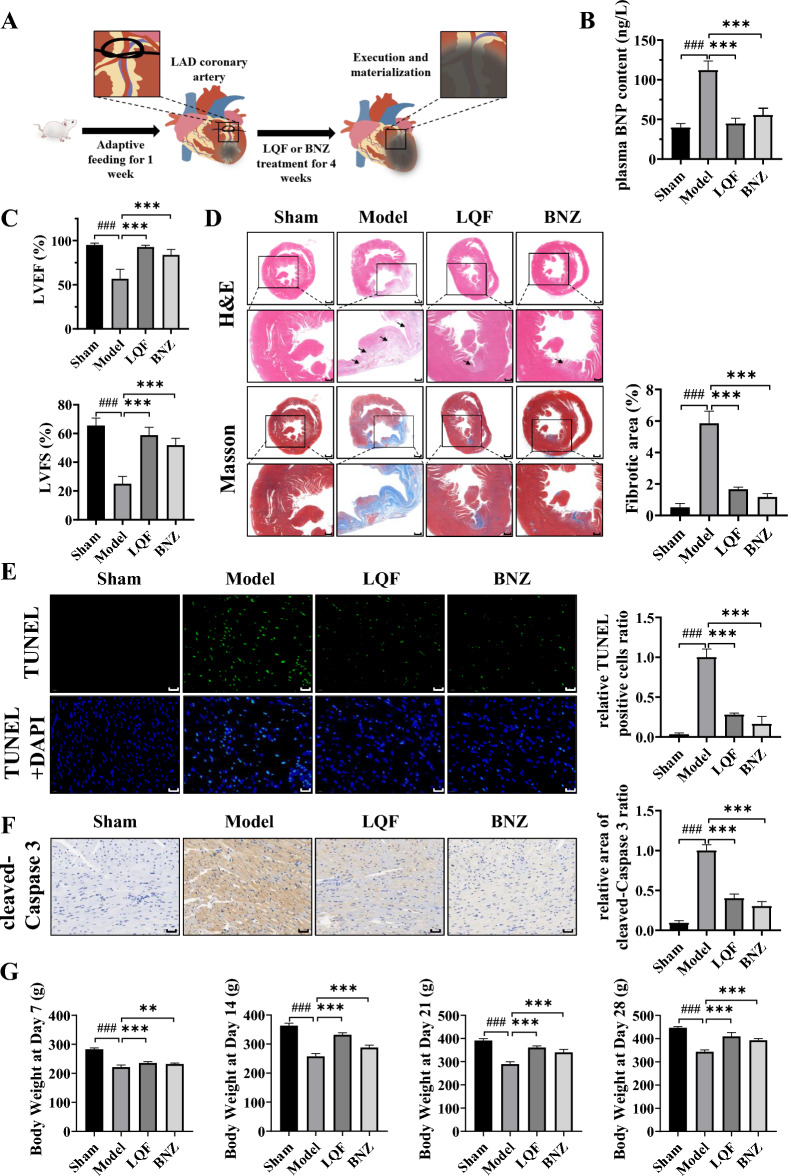
LQF relieves ventricular remodeling after AMI in vivo. **A** Ventricular remodeling was constructed in Wistar rats and randomly divided into 4 groups (for all groups, n = 6). Rats were administered by gavage with LQF (dry powder, 315 mg/kg, equal to clinical dose for human) or BNZ (10 mg/kg) for 4 weeks. **B** Plasma BNP content (ng/L). **C** Cardiac function indexes LVEF and LVFS of rats were measured with echocardiography. **D** H&E and Masson’s trichrome staining of the infarcted area of heart (scale bar = 1 mm, and for the zoom, scale bar = 0.5 mm). Three views were randomly selected from each slice (n = 6 for each group) and the blue collagen area of each slice was quantitatively analyzed using ImageJ software. The average blue collagen area of the model group was calibrated to a value ‘one’, and the remaining groups were analyzed relative to the model group. **E** TUNEL staining of the infarcted area of heart (scale bar = 20 μm). Three views were randomly selected from each slice (n = 6 for each group) and fluorescence intensity of each slice was quantitatively analyzed using ImageJ software. The average fluorescence intensity of the model group was calibrated to a value ‘one’, and the remaining groups were analyzed relative to the model group. **F** Immunohistochemistry of the infarcted area of heart (scale bar = 50 μm) Three views were randomly selected from each slice (n = 6 for each group) and the positive area of each slice was quantitatively analyzed using ImageJ software. The average positive area of the model group was calibrated to a value ‘one’, and the remaining groups were analyzed relative to the model group. **G** Body weight of ventricular remodeling rats after LQF, BNZ or vehicle treatment and sham rats were recorded every week during the intervention of the drug. All experimental data are expressed as mean ± standard deviation, ### *p* < 0.001 vs Sham, ** *p* < 0.01, *** *p* < 0.001, ns (not significant) compared with indicated group

Ventricular remodeling was induced in rats by permanently ligating the LAD coronary artery (Fig. 1A). After 4 weeks, the cardiac function of rats in the model group significantly deteriorated compared to the sham group. The model group exhibited a significant increase in plasma BNP level which was detected by ELISA (Fig. 1B). Echocardiography revealed a reduction in LVEF and LVFS in model group (Fig. 1C). Following a four-week treatment with LQF and BNZ (an angiotensin-converting enzyme inhibitor commonly used to treat HF), which served as positive control, there was a significant improvement in cardiac function in rats. Furthermore, levels of BNP, LVEF and LVFS after LQF treatment were nearly restored to those observed in the sham group (Fig. 1B, C). H&E and Masson’s trichrome staining indicated myocardial hypertrophy and collagen deposition in the model group, which were improved after LQF or BNZ administration (Fig. 1D). In addition, LQF treatment inhibited cardiomyocyte apoptosis in the infarct areas. TUNEL assay showed that the proportion of apoptotic cardiomyocytes significantly reduced after treatment of LQF and BNZ (Fig. 1E). Consistently, the expression of cleaved-Caspase 3 in myocardial tissue was also decreased by LQF and BNZ (Fig. 1F). Furthermore, the weight gain rate of rats in the model group slowed down significantly but was markedly improved by LQF administration (Fig. 1G). These findings suggest that LQF attenuates ventricular remodeling and improves cardiac function in rats.

### LQF attenuates intestinal barrier injury induced by ventricular remodeling

**Fig. 2:**
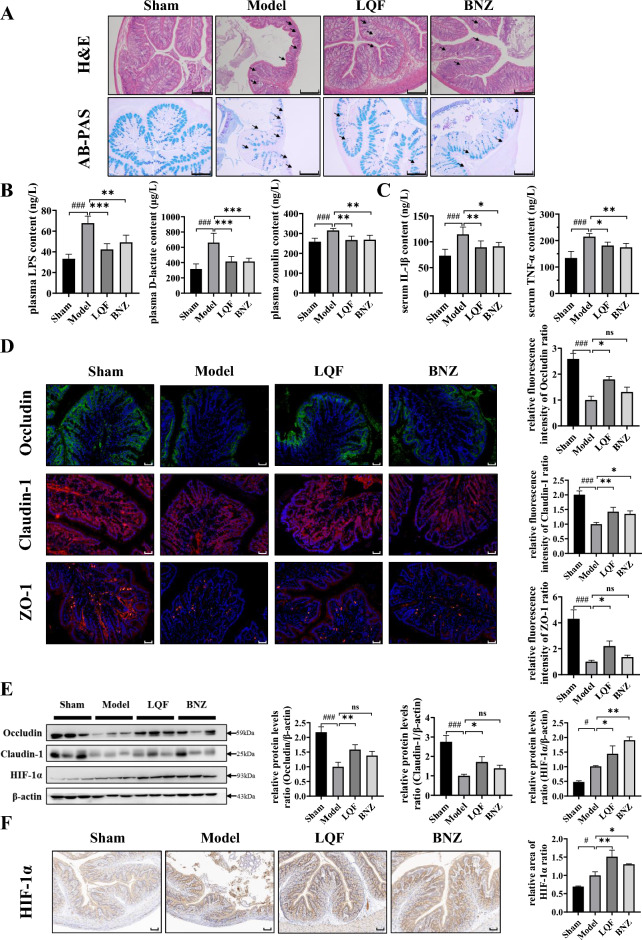
LQF maintains intestinal barrier integrity in ventricular remodeling rats. **A** H&E (scale bar = 200 μm) and AB-PAS staining (scale bar = 200 μm) of colon (for all groups, n = 6). **B** Plasma LPS (ng/L), D-lactate (μg/L) and zonulin (ng/L) content in rats. **C** Serum TNF-α (ng/L), IL-1β (ng/L) content in rats. **D** Immunofluorescence of Occluding, Claudin-1, ZO-1 of colon (scale bar = 50 μm). Three views were randomly selected from each slice (n = 6 for each group) and the fluorescence intensity of each slice was quantitatively analyzed using ImageJ software. The average fluorescence intensity of the model group was calibrated to a value ‘one’, and the remaining groups were analyzed relative to the model group. **E** Protein expression of Occluding, Claudin-1 and HIF-1α of colon. β-actin served as a loading control. Three samples were randomly selected from each group for target protein detection, and the grayscale values of each band were quantitatively analyzed using ImageJ software. The average grayscale value of the model group was calibrated to a value ‘one’, and the remaining groups were analyzed relative to the model group. **F** Immunohistochemistry of HIF-1α of colon (scale bar = 50 μm). Three views were randomly selected from slice (n = 6 for each group) and the positive area of each slice was quantitatively analyzed using ImageJ software. The average positive area of the model group was calibrated to a value ‘one’, and the remaining groups were analyzed relative to the model group. All experimental data are expressed as mean ± standard deviation, # *p* < 0.05, ## *p* < 0.01, ### *p* < 0.001 vs Sham, * *p* < 0.05, ** *p* < 0.01, *** *p* < 0.001, ns (not significant) compared with indicated group

To investigate the potential of LQF treatment in ameliorating intestinal barrier injury induced by ventricular remodeling, we conducted further analyses on the intestinal permeability in rats of different groups. Compared with the sham group, rats subjected to four weeks of LAD ligation exhibited distorted colonic crypts, severe epithelial damage, increased inflammatory cell infiltration, and reduced goblet cells, indicating compromised intestinal barrier (Fig. 2A). The histological findings confirmed that the defective intestinal barrier observed in the model group was associated with elevated circulating concentrations of microbiota-derived metabolites, such as LPS, D-lactate and zonulin (Fig. 2B). Meanwhile, LQF treatment significantly improved the gut permeability and attenuated circulatory elevation of LPS, D-lactate and zonulin levels in ventricular remodeling rats. Furthermore, LQF also attenuated the increased expression of the systemic inflammatory cytokines (TNF-α, IL-1β) in rats induced by ventricular remodeling (Fig. 2C). Notably, compared to the model group, the efficacy of LQF in improving intestinal barrier injury is much better than that of BNZ at the indicated dosage.

To further elucidate the mechanisms underlying the beneficial effects of LQF on the intestinal barrier integrity, we examined the expression of TJ proteins, which play a crucial role in maintaining the epithelial barrier integrity of intestine, such as Occludin, Claudin-1 and ZO-1 [31]. LQF significantly restored the decreased expression of these intestinal TJ proteins in the ventricular remodeling rats (Fig. 2D, E). HIF-1α activation has been implicated in the protective effect on the intestinal barrier [32], partially by regulating TJ proteins [21, 33]. As a result, HIF-1α protein expression and nuclear translocation were elevated in colon tissue in response to the hypoxia stress induced by ventricular remodeling, and further markedly increased by LQF treatment (Fig. 2E, F and Additional file 1: Figure S2). Together, these results suggested that LQF remarkably alleviated the intestinal barrier injury induced by ventricular remodeling, and HIF-1α may play an important role in the action of LQF.

### LQF maintains intestinal barrier integrity by upregulating HIF-1α in colon

**Fig. 3:**
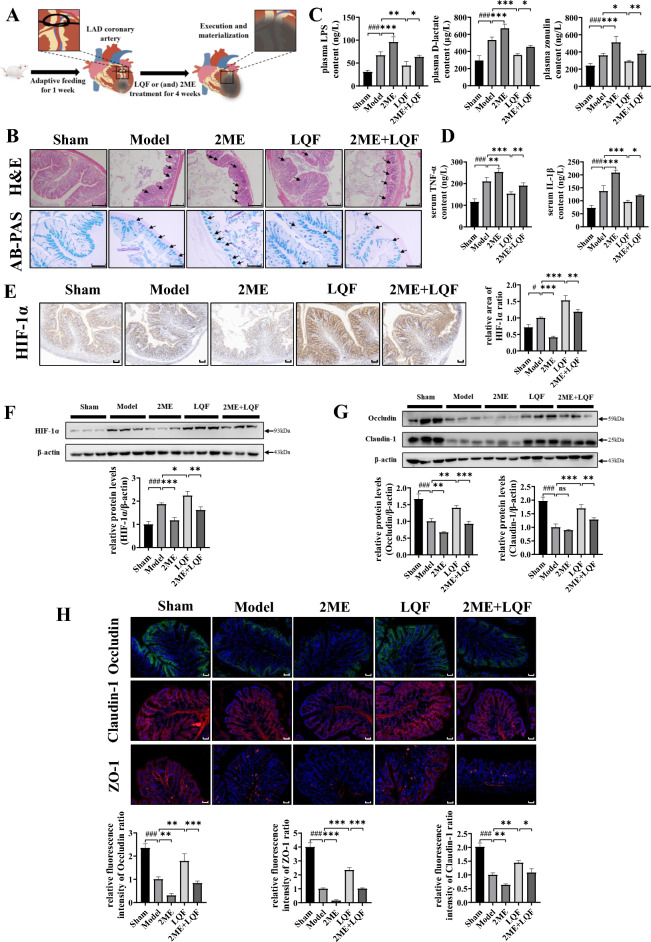
LQF maintains intestinal barrier integrity by upregulating HIF-1α in colon. **A** Ventricular remodeling was constructed in Wistar rats and randomly divided into 4 operated groups, and set up a sham-operated group as a control (for 2ME group, n = 4, 2ME + LQF group, n = 5, other groups, n = 6). Rats were administrated by gavage with LQF alone (315 mg/kg) or intraperitoneally administrated with 2ME (15 mg/kg) or both for 4 weeks. **B** H&E (scale bar = 200 μm) and AB-PAS staining (scale bar = 200 μm) of the colon. **C** Plasma LPS (ng/L), D-lactate (μg/L), and zonulin (ng/L) content in rats were measured. **D** Serum TNF-α (ng/L), and IL-1β (ng/L) content in rats were measured. **E**, **F** Immunohistochemistry (scale bar = 50 μm) and protein expression of HIF-1α in the colon were shown. Three views were randomly selected from each slice (for 2ME group, n = 4, 2ME + LQF group, n = 5, other groups, n = 6) for target protein detection, the fluorescence intensity and grayscale values were quantitatively analyzed using ImageJ software. The fluorescence intensity and grayscale values of the model group were calibrated to a value ‘one’, respectively, and the remaining groups were analyzed relative to the model group. **G** Protein expression of Occluding and Claudin-1 in the colon. β-actin served as a loading control. Three samples were randomly selected from each group for target protein detection, and the grayscale values of each band were quantitatively analyzed using ImageJ software. The average grayscale value of the model group was calibrated to a value ‘one’, and the remaining groups were analyzed relative to the model group. **H** Immunofluorescence of Occludin, Claudin-1, and ZO-1 in the colon (scale bar = 50 μm). Three views were randomly selected from each slice (for 2ME group, n = 4, 2ME + LQF group, n = 5, other groups, n = 6) and the fluorescence intensity of each slice was quantitatively analyzed using ImageJ software. The average fluorescence intensity of the model group was calibrated to a value ‘one’, and the remaining groups were analyzed relative to the model group. All data are expressed as mean ± standard deviation, # *p* < 0.05, ### *p* < 0.001, vs Sham, * *p* < 0.05, ** *p* < 0.01, *** *p* < 0.001, ns (not significant) compared with indicated group

Ventricular remodeling leads to hypoxic blood circulation, resulting in a hypoxic environment in the colon where HIF-1α plays a protective role [34, 35]. To verify the role of HIF-1α in the effect of LQF on maintaining intestinal barrier integrity, 2ME, a HIF-1α inhibitor was intraperitoneally administrated to ventricular remodeling rats, with or without LQF treatment by gavage for 4 weeks (Fig. 3A). The destructive influence of 2ME on the colon barrier led to reduced TJ proteins expression, increased intestinal permeability and systemic inflammation, suggesting the important regulatory function of HIF-1α in maintaining intestinal barrier integrity (Fig. 3B–H). Morphology analysis revealed that 2ME weakened the protective effects of LQF against colonic crypts distortion, severe epithelial damage, inflammatory cell infiltration and goblet cells reduction caused by ventricular remodeling (Fig. 3B). Consistently, LQF's effect in reducing the levels of LPS, D-lactate, zonulin, TNF-α and IL-1β in blood, as well as HIF-1α expression and nuclear translocation in colon tissue, was significantly rescued by 2ME (Fig. 3C–F and Additional file 1: Fig. S2). The increased expression of TJ proteins including Occludin, Claudin-1 and ZO-1 were also diminished by 2ME supplementation (Fig. 3G, H). Consistent with the in vivo results, exposure of caco-2 cells to hypoxic stress led to increased expression of HIF-1α and decreased expression of Occludin and Claudin-1. Treatment with LQF significantly protected the expression of Occludin and Claudin-1 and increased HIF-1α expression. However, this protective effect of LQF on Occludin and Claudin-1 expression was significantly attenuated by the addition of 2ME, as shown in (Additional file 1: Fig. S3). Overall, we demonstrated that LQF at least partially maintained intestinal barrier integrity by up-regulating HIF-1α protein expression.

### LQF relieves ventricular remodeling through HIF-1α-mediated intestinal barrier integrity

**Fig. 4:**
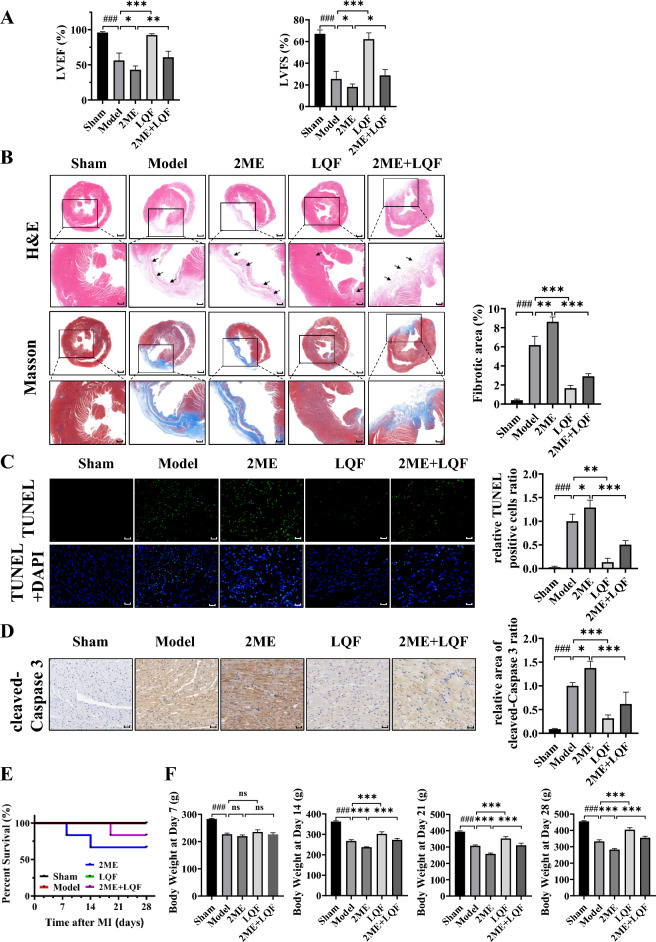
LQF relieves ventricular remodeling partially through HIF-1α-mediated intestinal barrier integrity. **A** Cardiac function indexes, LVEF and LVFS levels of ventricular remodeling rats were measured with echocardiography (for 2ME group, n = 4, 2ME + LQF group, n = 5, other groups, n = 6). **B** H&E and Masson’s trichrome staining of the infarcted area of heart (scale bar = 1 mm, and for the zoom, scale bar = 0.5 mm). Three views were randomly selected from each slice (for 2ME group, n = 4, 2ME + LQF group, n = 5, other groups, n = 6) and the blue collagen area of each slice was quantitatively analyzed using ImageJ software. The average blue collagen area of the model group was calibrated to a value ‘one’, and the remaining groups were analyzed relative to the model group. **C** TUNEL of the infarcted area of heart (scale bar = 20 μm). Three views were randomly selected from each slice (for 2ME group, n = 4, 2ME + LQF group, n = 5, other groups, n = 6) and the fluorescence intensity of each slice was quantitatively analyzed using ImageJ software. The average fluorescence intensity of the model group was calibrated to a value ‘one’, and the remaining groups were analyzed relative to the model group. **D** Immunohistochemistry of the infarcted area of heart (scale bar = 50 μm). Three views were randomly selected from each slice (for 2ME group, n = 4, 2ME + LQF group, n = 5, other groups, n = 6) and the positive area of each slice was quantitatively analyzed using ImageJ software. The average positive area of the model group was calibrated to a value ‘one’, and the remaining groups were analyzed relative to the model group. **E**, **F** Rats’ survival rates were recorded and the changes in body weight were recorded every week. All experimental data are expressed as mean ± standard deviation, ### *p* < 0.001 vs Sham, * *p* < 0.05, ** *p* < 0.01, *** *p* < 0.001, ns (not significant) compared with indicated groups

Since the “heart-gut axis” in ventricular remodeling rats forms a feedback regulatory loop [10], we then determined whether the protective effect of LQF on HIF-1α-mediated intestinal barrier integrity might eventually improve cardiac function. As shown in Fig. 4A, 2ME exacerbated cardiac dysfunction in ventricular remodeling rats as evidenced by decreased levels of LVEF and LVFS. However, LQF treatment was able to alleviated cardiac dysfunction. Similar results were observed in heart morphology, where collagen fiber deposition and myocardial fibrosis were further exacerbated by 2ME, but effectively attenuated after LQF treatment (Fig. 4B). Cardiomyocyte apoptosis induced by ventricular remodeling was further provoked by 2ME treatment, but significantly suppressed by LQF (Fig. 4C, D). Additionally, the loss of HIF-1α led to slower weight gain and a lower survival rate in 2ME group, which was partially rescued by LQF, indicating that LQF reversed the injury caused by 2ME treatment in rats with ventricular remodeling (Fig. 4E, F). Taken together, intestinal barrier damage caused by HIF-1α reduction aggravated ventricular remodeling, which could be alleviated by LQF.

### LQF improves cardiac function and intestinal barrier integrity in patients

**Fig. 5:**
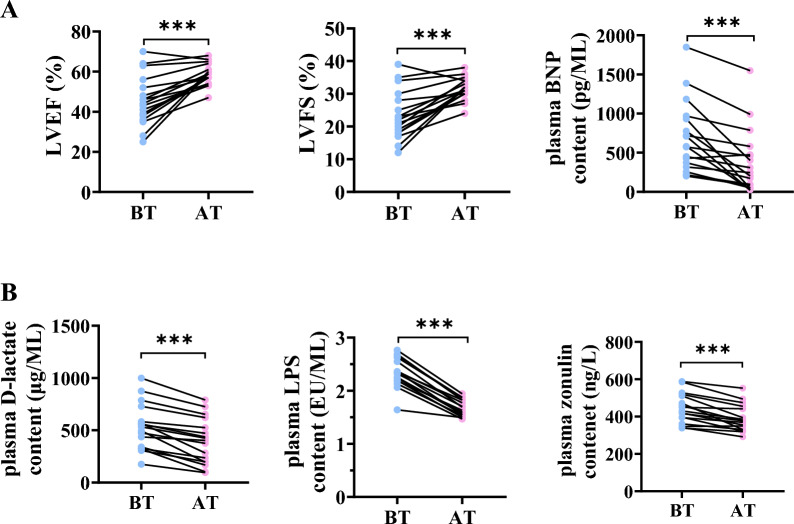
LQF protects cardiac function potentially by regulating intestinal barrier integrity. **A** The cardiac function indexes LVEF, LVSV and plasma BNP content (pg/mL) of HF patients before and after LQF treatment (oral treatment for 2–4 months, with a daily dose equivalent to 107 g of raw drug). **B** Plasma LPS (EU/mL), D-lactate (μg/mL) and zonulin (ng/L) content of HF patients. * *p* < 0.05, *** *p* < 0.001 vs before treatment. n = 17

To further validate the clinical efficacy of LQF on cardiac function and intestinal barrier integrity, 17 patients with HF who met the inclusion criteria were admitted to our hospital and treated with LQF. (Table 1). We examined clinical indicators of cardiac function, such as LVEF, LVFS, LVSV, LVEDV and LVESV, as well as the HF biomarker BNP. The results showed that LQF treatment lasting 2–4 months, with a daily dose equivalent to 107 g of raw drug, significantly improved cardiac function, and alleviated the progression of HF (Table 2, Fig. 5A). Intestinal permeability markers, including LPS, D-lactate and zonulin were found decreased after LQF treatment, suggesting that maintaining intestinal barrier integrity may contribute to the improvement of cardiac function in HF patients (Table 2, Fig. 5B).

**Table 1:** Demographic data of patients with HF taking LQF

Variables	Patients ( n = 17)
Age ( year)	
≤ 50	3 ( 17.6%)
50–80	9 ( 52.9%)
≥ 80	5 ( 29.4%)
Gender	
Male	13 ( 76.4%)
Female	4 ( 23.5%)
NYHA Grade	
Grade II	9 (52.9%)
Grade III	5 (29.4%)
Grade IV	3 (17.6%)

**Table 2:** Patients' clinical progress before and after treatment of LQF

Parameters	Before treatment	After treatment	P-value
LVEF (%)	45.53 ± 3.009	58.47 ± 1.289	< 0.0001***
LVFS (%)	23.41 ± 1.833	31.53 ± 0.8275	< 0.0001***
LVSV (logmL)	1.807 ± 0.04616	1.922 ± 0.03816	0.0195*
LVEDV (logmL)	2.198 ± 0.04086	2.149 ± 0.04484	0.1731
LVESV (logmL)	1.931 ± 0.0611	1.814 ± 0.04487	0.0560
ALT (U/L)	25.18 ± 2.801	22.38 ± 1.839	0.2670
AST (U/L)	24.5 ± 1.966	22.38 ± 1.363	0.2399
Carbamide (mmol/L)	6.831 ± 0.4997	7.278 ± 0.5945	0.5486
Creatinine (μmol/L)	89.4 ± 4.841	87.59 ± 6.965	0.7522
BNP (pg/mL)	701.6 ± 109.5	381.8 ± 99.67	0.0002***
LPS (EU/mL)	2.331 ± 0.06663	1.674 ± 0.03716	< 0.0001***
D-lactate (μg/L)	533.5 ± 52.85	394.8 ± 52.62	< 0.0001***
zonulin (ng/L)	444.5 ± 19.49	389.3 ± 17.31	< 0.0001***

All experimental data are expressed as mean ± standard deviation, * *p* < 0.05, *** *p* < 0.001 compared with before treatment. n = 17

*LVEF* left ventricular ejection fraction; *LVFS* left ventricular fractional shortening; *LVSV* left ventricular stroke volume; *LVEDV* left ventricular end-diastolic dimension; *LVESV* left ventricular end-systolic dimension; *ALT* Alanine transaminase; *AST* Aspartate aminotransferase; *BNP* brain natriuretic peptide; *LPS* endotoxin

## Discussion

Cardiac dysfunction can lead to intestinal ischemia and hypoxia, which in turn increases intestinal permeability and circulating inflammatory factors [7, 10]. Patients with HF exhibit an abnormally high abundance of pathogenic bacteria in their intestines, resulting in a 210% increase in colonic permeability compared to healthy individuals [36, 37]. This compromised intestinal barrier further exacerbates HF, revealing a complex interplay between the heart and gut, commonly referred to as the heart-gut axis. In our study, we demonstrated that LQF attenuates ventricular remodeling and improves cardiac function in both HF rats and patients. High intestinal barrier permeability was observed in rats and patients experiencing ventricular remodeling, which could be improved through LQF treatment. Furthermore, our findings showed that a compromised intestinal barrier further aggravated systemic inflammation and cardiac function, but these effects were alleviated by LQF treatment, indicating a potential role for the heart-gut axis in LQF’s therapeutic action.

To explore the underlying mechanism, we investigated whether LQF could act on HIF-1α, which plays a protective role in maintaining the physiological structure and function of the intestinal barrier under hypoxic stress conditions [34, 35]. We found that LQF improved cardiac function at least partially by maintaining HIF-1α-mediated intestinal integrity in rats and patients with HF. HIF-1α inhibitor treatment led to deterioration of intestinal integrity, which was rescued by LQF treatment. However, the improvement of intestinal barrier integrity brought by LQF did not completely restore cardiac function, suggesting that maintaining intestinal barrier integrity only partially contributed to the enhancement in cardiac function. Further studies are needed to elucidate the specific interactions and mechanisms within the loop of the heart-gut axis.

Currently, HF drug treatment primarily consists of medications like ACE inhibitors, beta-blockers, diuretics, and aldosterone antagonists, which help manage symptoms and reduce the risk of hospitalizations [38, 39]. These medications have proven effective in enhancing patients’ quality of life and extending survival rates. However, drug treatments may cause side effects or be less effective in some patients, underscoring the necessity for personalized therapy and the exploration of novel treatment options [40]. LQF offers several advantages for HF treatment by providing a holistic approach that addresses multiple aspects of the disease, including attenuating heart and kidney yang deficiency and inhibiting myocardial cell hypertrophy [41]. As a traditional Chinese medicine, LQF has fewer side effects and drug interactions compared to chemical drugs, making it a potentially safer option for long-term use. Its multifaceted approach targets the heart-gut regulatory axis, allowing for a more effective therapeutic intervention. The natural source of LQF offers a favorable safety profile, which enhances patient compliance and treatment outcomes. Additionally, LQF’s versatility enables its use with other treatments, promoting synergistic effects and improvements in cardiac function.

Although our study identified HIF-1α-mediated intestinal barrier integrity as one of potential mechanisms of LQF, many questions still remain unclear. For instance, the signaling pathways responsible for LQF-mediated upregulation of HIF-1α in colon tissue and the interactions between cell death signaling of cardiomyocyte warrant further investigation. Additionally, it is essential to conduct activity-based separation analyses to identify the bioactive compounds of LQF, which would facilitate target identification and ultimately contribute to modern prescription development [42]. Future research should explore these questions and further elucidate LQF's underlying mechanisms in HF treatment, thereby refining and enhancing current therapeutic approaches for patient benefit.

## Conclusion

In conclusion, our study demonstrated that LQF effectively protects cardiac function, in part by preserving intestinal barrier integrity in ventricular remodeling rats and patients. This protective effect was partially mediated by the upregulation of HIF-1α expression in colon tissue. The notable improvement in cardiac function and decreased circulating levels of intestinal permeability markers in HF patients emphasize LQF’s therapeutic potential. These findings contribute to a better understanding of the heart-gut regulatory axis and provide empirical evidence to support LQF’s clinical application.

## Supplementary Information

**Additional file1: Fig. S1. **The composition and quality control of LQF. UPLC-Q-TOF/MS showed that LQF contains seven pharmacopoeia reference substances (a) hydroxysafflor yellow A, (b) calycosin-7-O-beta-D-glucoside, (c) quercetin, (d) cinnamic acid, (e) astragaloside IV, (f) formononetin, (g) atractylenolide-1, as evidenced by comparing the peaks’ retention times between LQF solution and the reference substances. **Fig. S2. **LQF improved the nuclear translocation of HIF-1α in colon tissue. Immunohistochemistry (scale bar = 20 μm) of HIF-1α in the colon (for 2ME group, n = 4, 2ME + LQF group, n = 5, other groups, n = 6). Relative HIF-1α expression levels in nuclear were quantitatively analyzed using ImageJ software. All experimental data are expressed as mean ± standard deviation, ## *p*<0.01, ### *p*<0.001 vs Sham, ** *p*<0.01, *** *p*<0.001 compared with indicated groups. **Fig. S3.** LQF protected Occludin and Claudin-1 in hypoxic caco-2 cells by up-regulating HIF-1α. Protein expression of HIF-1α, Occludin and Claudin-1 in the caco-2 cell under hypoxia and treatment with 200 μg/mL LQF, 5 μM 2ME (HIF-1α inhibitor) or combination for 48 h. β-actin served as a loading control. The grayscale values of each band were quantitatively analyzed using ImageJ software, data are expressed as mean ± standard deviation of triplicate independent experiments. ### *p*<0.001, vs no hypoxic cell, ** *p*<0.01, *** *p*<0.001 compared with indicated group.

## Data Availability

All data generated or analyzed during this study are included in this published article.

## Abbreviations

AMI: Acute myocardial infarction
HF: Heart failure
TJ: Tight junction
ZO-1: Zonula occludens-1
LPS: Endotoxin
LQF: LuQi Formula
BNP: Brain natriuretic peptide
LVEF: Left ventricular ejection fraction
LVFS: Left ventricular fractional shortening
NLRP3: The Nod-like receptor family pyrin domain containing 3
TMAO: Trimethylamine N-oxide
HIF-1α: Hypoxia inducible factor-1 alpha
BNZ: Benazepril hydrochloride
2ME: 2-Methoxyestradiol
LAD: Left anterior descending branch
ELISA: Enzyme-linked immunosorbent assay
TNF-α: Tumor necrosis factor alpha
IL-1β: Interleukin-1 beta
HRP: Horseradish peroxidase
H&E: Hematoxylin–eosin
AB-PAS: Alcian Blue Periodic acid Schiff
TUNEL: TdT-mediated dUTP Nick-End Labeling
LVSV: Left ventricular stroke volume
LVEDV: Left ventricular end-diastolic dimension
LVESV: Left ventricular end-systolic dimension
ALT: Alanine transaminase
AST: Aspartate aminotransferase
