# Supplementary material for: LuQi Formula relieves ventricular remodeling through improvement of HIF-1α-mediated intestinal barrier integrity
Source: Chin Med. 2023 Jul 28;18:90. doi: 10.1186/s13020-023-00803-y (PMC10386699; doi:10.1186/s13020-023-00803-y)
Supplement: Supplementary file 1 — Additional file1: Fig. S1. The composition and quality control of LQF. UPLC-Q-TOF/MS showed that LQF contains seven pharmacopoeia reference substances (a) hydroxysafflor yellow A, (b) calycosin-7-O-beta-D-glucoside, (c) quercetin, (d) cinnamic acid, (e) astragaloside IV, (f) formononetin, (g) atractylenolide-1, as evidenced by comparing the peaks’ retention times between LQF solution and the reference substances. Fig. S2. LQF improved the nuclear translocation of HIF-1α in colon tissue. Immunohistochemistry (scale bar = 20 μm) of HIF-1α in the colon (for 2ME group, n = 4, 2ME + LQF group, n = 5, other groups, n = 6). Relative HIF-1α expression levels in nuclear were quantitatively analyzed using ImageJ software. All experimental data are expressed as mean ± standard deviation, ## p<0.01, ### p<0.001 vs Sham, ** p<0.01, *** p<0.001 compared with indicated groups. Fig. S3. LQF protected Occludin and Claudin-1 in hypoxic caco-2 cells by up-regulating HIF-1α. Protein expression of HIF-1α, Occludin and Claudin-1 in the caco-2 cell under hypoxia and treatment with 200 μg/mL LQF, 5 μM 2ME (HIF-1α inhibitor) or combination for 48 h. β-actin served as a loading control. The grayscale values of each band were quantitatively analyzed using ImageJ software, data are expressed as mean ± standard deviation of triplicate independent experiments. ### p<0.001, vs no hypoxic cell, ** p<0.01, *** p<0.001 compared with indicated group. [file 13020_2023_803_MOESM1_ESM.docx]

**Methods**

***HPLC-Q-TOF/MS analysis***

The components of LQF were detected by UPLC-Q-TOF/MS. MS analysis was performed on a Waters Q-TOF SYNAPT G2 mass spectrometer (Waters MS Technologies, Manchester, UK) equipped with an electrospray ionization source. Separation was performed on a Waters ACQUTTY HSS T3 column (100 mm × 2.1 mm, 1.8 μm) equipped with an online filter that was eluted with a binary mobile phase of (A) 0.1% formic acid in water and (B) 0.1% formic acid in acetonitrile as follows: linear gradient from 5 to 100% B (0-25 min). Data acquisition was controlled using MassLynx V4.2 software (Waters Corporation, Milford, USA). Leucine enkephalin was used as a lock mass. Uncalibrated continuum data were acquired for each sample from 100 to 1500 Da.

***Caco-2 cell culture and hypoxia model***

The caco-2 cell line was cultured in high -glucose Dulbecco’s modified Eagle’s medium containing 10% fetal bovine serum and 1% antibiotics penicillin/streptomycin in an incubator at 37℃ with 5% CO2. The medium was changed every 2-3 days. In the hypoxia models, cells were exposed to a chamber containing an activated carbon bag, which reacts with and absorbs oxygen. The cells were incubated with LQF (200 μg/ml), 2ME (5 μM), or a combination of both for 48 hours.

**Results**

**Fig. S1**


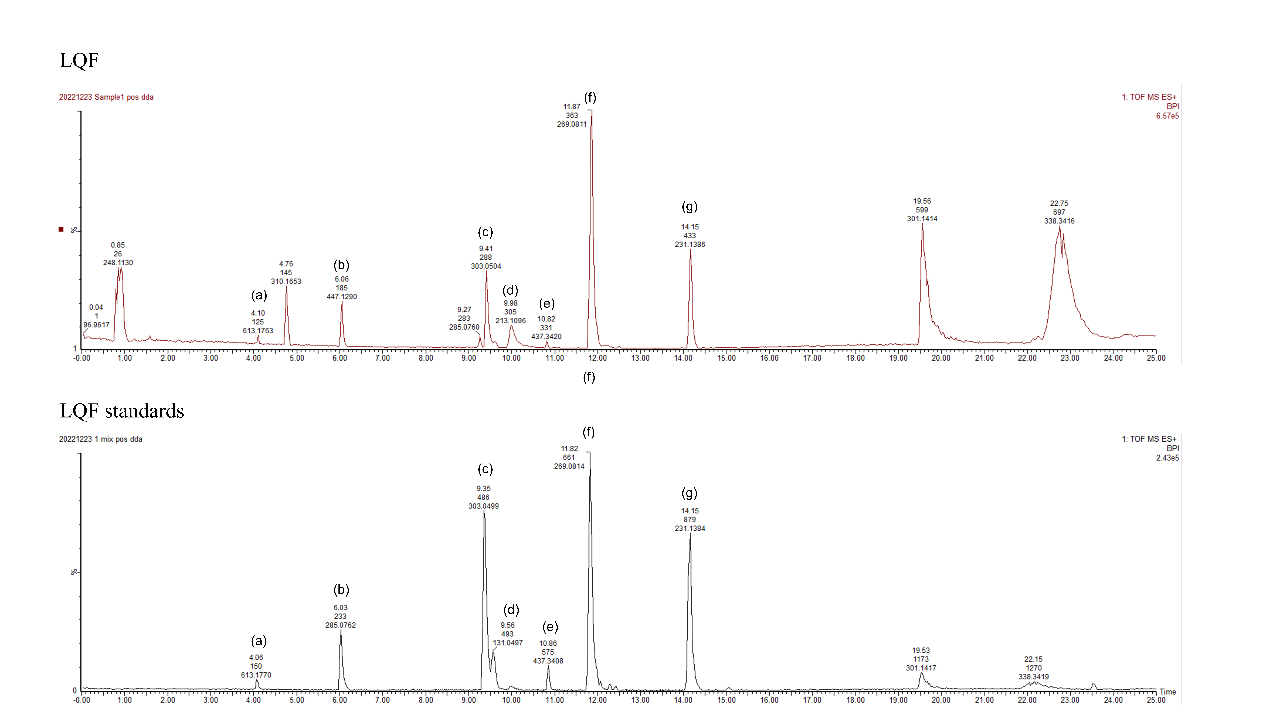


**Fig. S1. The composition and quality control of LQF.** UPLC-Q-TOF/MS showed that LQF contains seven pharmacopoeia reference substances (a) hydroxysafflor yellow A, (b) calycosin-7-O-beta-D-glucoside, (c) quercetin, (d) cinnamic acid, (e) astragaloside IV, (f) formononetin, (g) atractylenolide-1, as evidenced by comparing the peaks’ retention times between LQF solution and the reference substances.

**Supplementary Fig. 2**


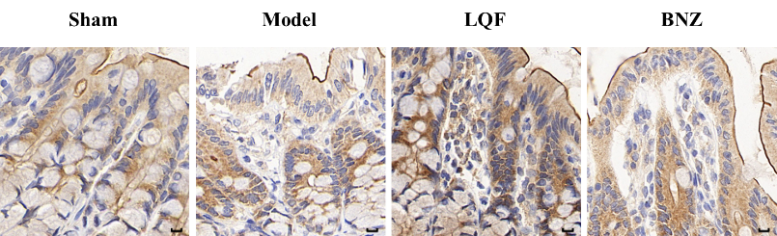


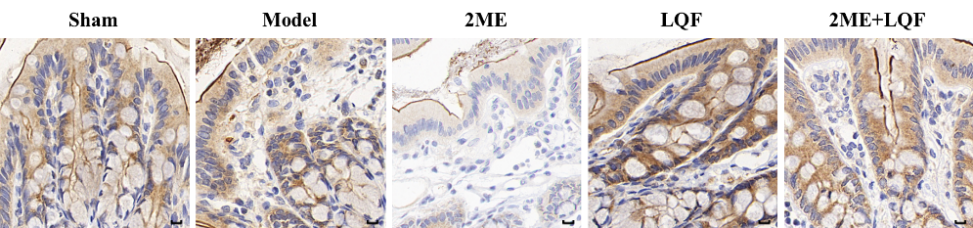


**Fig. S2. LQF improved the nuclear translocation of HIF-1α in colon tissue.** Immunohistochemistry (scale bar = 20 μm) of HIF-1α in the colon (for 2ME group, n = 4, 2ME + LQF group, n = 5, other groups, n = 6). Relative HIF-1α expression levels in nuclear were quantitatively analyzed using ImageJ software. All experimental data are expressed as "mean ± standard deviation", ## *p*<0.01, ### *p*<0.001 vs Sham, ** *p*<0.01, *** *p*<0.001 compared with indicated groups.

**Fig. S3.**


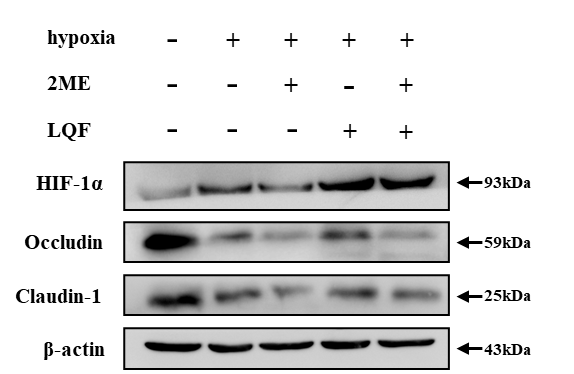


**Fig. S3.** **LQF protected** **Occludin and Claudin-1 in hypoxic caco-2 cells by up-regulating HIF-1α.** Protein expression of HIF-1α, Occludin and Claudin-1 in the caco-2 cell under hypoxia and treatment with 200μg/ml LQF, 5μM 2ME (HIF-1α inhibitor) or combination for 48 h. β-actin served as a loading control. The grayscale values of each band were quantitatively analyzed using ImageJ software, data are expressed as "mean ± standard deviation" of triplicate independent experiments. ### *p*<0.001, vs no hypoxic cell, ** *p*<0.01, *** *p*<0.001 compared with indicated group.
